# Effect of egg consumption on early childhood development: evidence from *Un Oeuf* study

**DOI:** 10.1017/S1368980024002490

**Published:** 2024-12-12

**Authors:** Helen Ernyey, Chhavi Tiwari, Heather Stark, Emma Hunniford, Aissata Wereme N’Diaye, Yacouba Zare, Anteneh Omer, Sarah Lindley McKune

**Affiliations:** 1 College of Medicine, University of Florida, Gainesville, FL, USA; 2 Department of Environmental and Global Health, College of Public Health and Health Professions, University of Florida, Gainesville 32611-7011, FL, USA; 3 Department of Epidemiology, College of Public Health and Health Professions, University of Florida, Gainesville, FL, USA; 4 Laboratoire de Recherche en Production et Santé Animales (LaRePSA), Institut de l’Environnement et de Recherches Agricoles (INERA), Centre National de Recherches Scientifique et Technologique–(CNRST), Ouagadougou, Burkina Faso; 5 Independent Nutrition Consultant, Hawassa, Ethiopia; 6 The Center for African Studies, University of Florida, Gainesville, FL, USA

**Keywords:** Early childhood development, Ages and Stages Questionnaire-3, Clustered randomised controlled trial, Eggs, Animal source food, Infant and child nutrition, Low-income countries

## Abstract

**Objective:**

Recent studies have shown that inclusion of eggs in young children’s diet can help meet nutritional requirements associated with cognitive development. This study aims to investigate the effect of egg consumption on early childhood development using Ages and Stages Questionnaire-3 in Burkina Faso.

**Design:**

The study presented here uses data collected during a follow-up of the *Un Oeuf*-a three-arm clustered randomised controlled trial (RCT), conducted roughly 4 months after the end of the RCT.

**Setting:**

This research was conducted in eighteen rural villages within the Kaya Department of the Sanmatenga Province in Burkina Faso.

**Participants:**

Participants of this study include a total of 244 children aged between 18 and 33 months, with seventy-eight children in the full intervention group, eighty-three in the partial group, and eighty-three in the control group.

**Results:**

Results show that children with consistent egg consumption (in all months) had a lower odd of falling below the cut-off scores in gross motor ( 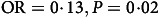 ) and personal social skills ( 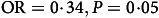 ). And a dose–response was established; for each additional egg/week, a 1·9 % increase in scores for problem-solving skills was observed.

**Conclusions:**

Findings from this study contribute to a growing body of evidence that increasing egg consumption among children in low- and middle-income countries (LMIC) can improve growth and development. The study highlights the need for additional research in LMIC to better understand the multifactorial relationship between diet and childhood development.

Early childhood development (ECD) was included in the UN’ Sustainable Development Goals in 2015, signifying a global commitment to ensure equitable access to early learning opportunities and childhood development for all children^(1)^. Early childhood offers a critical window of opportunity that can shape the lifetime trajectory of a child’s holistic development, including academic achievement, occupational success and social adjustment^(2,3)^. Delay in ECD is more common in low- and middle-income countries (LMIC), with 250 million children under five at risk of not achieving their developmental potential^(4)^, and around 42 % of children experiencing childhood delays living in West Africa^(5)^.

ECD is impacted by myriad factors, including diet, environmental, and social risk exposures^(6,7)^. One of the important underlying reasons for the high prevalence of ECD delays is underlying poor nutrition and food insecurity in LMIC, which emerges from lack of access to resources alongside inadequate and inequitable food production^(8)^. Undernutrition during childhood leads to adverse health effects, including stunting, decreased cognitive functioning, and increased morbidity and mortality^(7,9,10)^. In Burkina Faso, over 672 000 children suffer from chronic malnutrition,^(11)^ with 22·6 % children under 5 suffering from stunting^(12)^. The high prevalence of stunting in Burkina Faso raises significant concerns for policymakers and necessitates additional research on nutrition and ECD in this context.

While meeting the nutritional requirement of early age is a challenge in most LMIC due to lack of resources, access to nutritious food^(13)^ and suboptimal feeding practices^(14)^, it has been shown that including animal source foods such as eggs, meat, fish, or dairy in otherwise typical diets has the potential to improve a child’s nutritional and health outcomes^(15)^. However, the consumption of animal source foods in LMIC has remained low, likely due to poor availability, high relative cost^(16)^, and restrictive food norms and taboos^(17)^. Nutritional interventions and behaviour change campaigns have been shown to reduce or remove the social and cultural barriers to consuming certain food items that may significantly impact early childhood growth and development^(18–20)^.

Like other animal source foods, eggs are rich in macro and micronutrients^(21,22)^. Consuming one egg per day for a child aged 6–24 months can provide adequate nutrients that are essential for child nutrition and brain development^(23)^.

Based on evidence from Ecuador^(24)^ that found significant improvements in child growth when infants were fed eggs, the *Un Oeuf* study – a clustered randomised controlled trial – was conducted in eighteen rural villages of Burkina Faso in 2018–2019, with the primary objective of increasing egg consumption among infants and young children through a behaviour change communication intervention^(19)^. In Kaya Department, the *Un Oeuf* study randomly selected eighteen non-urban villages and then randomly assigned (1:1:1) the villages to one of the three intervention arms. In the full intervention arm, children were gifted three chickens from a community champion and a fourth chicken from their fathers and caregivers received a behaviour change communication (BCC) package, including monthly trainings on integrated nutrition and agriculture. At a gifting ceremony, where the child received the chickens, village leaders presented the chickens as gifts to the child’s flock, reinforcing an understanding that the chickens and any eggs produced belonged to the child. One egg per day from these chickens was advised to be fed to the child. While the project did not intervene on the sale or sharing of eggs, mothers were trained on the significance of diet during the first 1000 d and asked to see the egg as a treatment for or investment in the targeted child. They were also advised that only after the child’s one egg/day ‘dose’ had been achieved, might the mother consider feeding other children or selling the eggs. The partial intervention group received only the BCC package and did not receive any chickens. The control group did not receive any intervention; neither BCC nor chickens. The intervention, which included monthly BCC trainings for the full and partial intervention groups, continued for 9 months with baseline data collection in the month of July 2018 and the end line data collection in April 2019. The trial was registered at clinicaltrials.gov: NCT04135625. For a detailed protocol of the study design, methods and baseline characteristics, see Stark *et al.*, 2021. The *Un Oeuf* study population ate little to no eggs at baseline; however, the intervention significantly increased egg consumption in the full and partial groups; the full intervention, in which children consumed around six eggs per week by end line, also significantly decreased wasting and underweight^(19)^. As the *Un Oeuf* study significantly improved egg consumption and nutritional status of children, additional funding was secured to investigate the potential impact of the study on ECD. The study presented here uses data collected during a follow-up of the *Un Oeuf* study, conducted roughly 4 months after the end of the randomised controlled trial and engaging the same children who participated in the initial study. This study aims to investigate the effect of egg consumption on ECD using the Ages and Stages Questionnaire (ASQ-3; Squires & Bricker, 2009), modified by the study team for the local environment.

## Methods

### Study design

The follow-up study was conducted 4 months after the completion of the *Un Oeuf* study to assess the impact of egg consumption on ECD in Burkina Faso^(25)^. During the 9-month intervention period of the *Un Oeuf* study, monthly surveys were used to collect data on egg consumption and anthropometric measurements of enrolled children. At the beginning of the study, children of age 6–12 months were enrolled. Upon conclusion, these children were assessed for development outcomes in the follow-up study, using a culturally modified ASQ-3 tool and anthropometric measurements of height, length, weight and head circumference.

### Variables

#### Child development measures

ASQ-3 is a widely used standardised screening instrument to measure development progress in children between the ages of 1 month to 66 months ^(26–30)^. The research team selected and adapted the ASQ tool to assess ECD in Burkina Faso in collaboration with scientists at the Anita Zucker Center for Excellence in Early Child Development Studies. Guided by these experts, changes in the ASQ-3 tool were made to allow for appropriate assessment of an indicator, while utilising materials that were more familiar to children in the Burkinabe context. These included substituting a stick that the children could use to write/draw in the dirt, since they did not have access to pencils and papers; using small pebbles instead of cheerios and stepping onto logs instead of climbing steps. Researchers then trained the enumerators from the *Un Oeuf* study to use the modified ASQ-3 tool, after working with them to review and revise the proposed tool for cultural salience. The questionnaire was then pilot-tested with children of appropriate ages using set criteria established to assess the various questions to improve internal reliability and validity prior to administering the ASQ-3 assessment. The ASQ-3 assessment was verbally administered to the child’s mother. In accord with ASQ-3 instruction, the enumerator asked the mother to respond to the questions and, in some cases, asked the child to perform an activity. These data were recorded by the enumerator. ASQ items were observed by the enumerator or reported by a parent and consist of thirty items scored as *yes, sometimes,* or *not yet* across five domains: communication, gross motor, fine motor, problem-solving and personal social skills. Within each domain, individuals can score from 0 to 60, with specific scores above or below the cut-off for expected childhood development. We examined cut-off scores in each domain of development. These domain-specific cut-off scores align with ASQ-3 guidelines, which use ASQ score and age of the child to determine the cut-offs. A binary indicator was used to describe whether a child’s score was below the cutoff value (1) or not (0). Following methodological approach of previous ECD research^(27)^, we looked at domain-specific scores, as well as the overall total score (sum of the five domain-specific scores).

#### Egg consumption measures

After the baseline survey, the first 2 months’ data quality was compromised due to heavy rains and inaccessibility to the study site. This study, therefore, used longitudinal information for egg consumption for 7 months (months 3–9) with baseline variables measured at the beginning (month 0) and child development scores measured in the follow-up (at month 12). In each month of the intervention period (month 3 through month 9), respondents were asked if they fed eggs to the child in the previous month. We created a categorical variable for consistency in egg consumption as: *1 = Never, 2 = 1–3 months; 3 = 4–6 months and 4 = in all 7 months*. Further, if/when respondents indicated that eggs were fed to the child, they were also asked how many eggs were fed in the previous week. Based on their responses, an average weekly egg consumption is calculated using 7 months of data. The measure calculates on average, how many eggs the child ate per week in the last 7 months. Therefore, we check for the effect of both consistency over time and quantity of egg consumption on child development outcomes.

As child development scores were only assessed after the trial ended, the analysis adjusted available confounding factors at baseline that are identified as determinants of child development in existing literature^(31)^. These confounders include child’s gender, birth order, baseline weight and height, mother’s age at first birth, mother’s education, household size and economic status (wealth index). The wealth index was calculated based on possession of assets and quality of housing using principal component analysis following Demographic and Health Survey guidelines and treated as tercile for modelling purposes. The items in the asset score included flooring material, cooking fuel, electricity, radio, television, cellphone, table, chair, mattress, solar panel, lamp, cycle, bike and cart. Information for asset ownership was collected only in the seventh month of data collection. The study does not include child’s own age as a confounder since ages and stages accounts for the age itself in score as well as the cut-off values.

### Statistical analysis

All analysis was performed in STATA, v.17. Two separate regression analyses have been performed contingent on the nature of the dependent variable. First, logistic regressions were employed to calculate the odds of children falling below the domain-specific cut-off scores while accounting for potential confounders to egg consumption and child growth. Further, linear regression models were used for domain-specific scores and for the total scores. Logarithmic transformations of these scores were used to ensure normality in the distribution and to look at the percent change in scores due to egg consumption. In all models, se were clustered at village level to account for village-level randomisation and heterogeneity across villages. We also tested these models by including intervention arms, but since the intervention of the *Un Oeuf study* was specifically designed to increase egg consumption in children through behaviour change, gifting chickens and integrated nutrition and agriculture training, the intervention arms (full and partial) were found to be highly correlated with the egg consumption (correlation coefficients, *r* = 0·92 and *r* = 0·70, respectively), thereby, could not be included in the regression model. In addition, propensity score matching is used to ensure robustness of the results and deal with selection bias.

## Results

### Descriptive statistics

The final analysis included a total of 244 children aged between 18 and 33 months, with seventy-eight children in the full intervention group, eighty-three in the partial group, and eighty-three in the control group. Descriptive statistics for baseline characteristics, egg consumption and child development outcomes across the three research arms are presented in Table 1.

**Table 1.** Descriptive statistics by intervention groups

	All (*n* 244)			Full (*n* 78)			Partial (*n* 83)			Control (*n* 83)		
Baseline variables												
Sex of the child, % (*n*)	%	*n*		%	*n*		%	*n*		%	*n*	
Boy	52·05	127		56·41	44		44·58	37		55·42	46	
Girl	47·95	117		43·59	34		55·42	46		44·58	37	
	Mean	sd	95 % CI	Mean	sd	95 % CI	Mean	sd	95 % CI	Mean	sd	95 % CI
Birth order	3·42	1·87	3·2, 3·7	3·45	1·7	3·1, 3·8	3·51	1·94	3·1, 3·9	3·3	1·97	2·9, 3·7
Weight (in kg)	7·9	1·09	7·7, 8	7·92	1·34	7·6, 8·2	7·7	0·98	7·5, 7·9	8	0·9	7·8, 8·2
Height (in cm)	70·5	59·6	69·9, 70·9	70·97	4·45	69·9, 71·9	69·97	4·38	69·01, 70·9	70·5	3·9	69·6, 71·3
Mother’s age at first birth	18·2	2·1	17·9, 18·5	18·2	2·2	17·7, 18·7	18·5	2·1	18·1, 18·9	17·9	1·8	17·6, 18·4
Education of mothers, % (*n*)												
	%	*n*		%	*n*		%	*n*		%	*n*	
No formal education	79·8	194		75·64	59		76·83	63		86·75	72	
Early primary education	5·35	13		6·41	5		6·1	5		3·61	3	
Complimentary primary studies	4·94	12		5·13	4		6·1	5		3·61	3	
Early secondary education	5·76	14		6·41	5		6·1	5		4·82	4	
* Koranic* school	4·12	10		6·41	5		4·9	4		1·2	1	
HH size, % (*n*)												
Less than 10	36·07	88		26·92	21		39·76	33		40·96	34	
11–20 persons	31·56	77		42·31	33		28·92	24		24·10	20	
21–30 persons	17·62	43		16·67	13		15·66	13		20·48	17	
More than 30	14·75	36		14·10	11		15·66	13		14·46	12	
Wealth index^*^, % (*n*)												
Lowest	33·3	85		38·46	30		22·89	19		38·55	32	
Middle	33·3	85		32·05	25		33·73	28		34·94	29	
Highest	33·3	85		29·49	23		43·37	36		26·51	22	
Egg consumption during the trial												
	Mean	sd	95 % CI	Mean	sd	95 % CI	Mean	sd	95 % CI	Mean	sd	95 % CI
Average weekly egg consumption	2·72	2·7	2·4, 3·1	6·39	0·8	6·2, 6·6	1·76	0·09	1·5, 1·9	0·22	0·48	0·12, 2·33
Consistency in egg consumption, % (*n*)												
	%	*n*		%	*n*		%	*n*		%	*n*	
Never	12·7	31		0			0			37·35	31	
1–3 months	25·82	63		0			19·28	16		56·63	47	
4–6 months	26·64	65		7·69	6		66·27	55		4·82	4	
In all months	34·84	85		92·31	72		14·46	12		1·2	1	

* Captured only in month 7.

**Figure 1.**
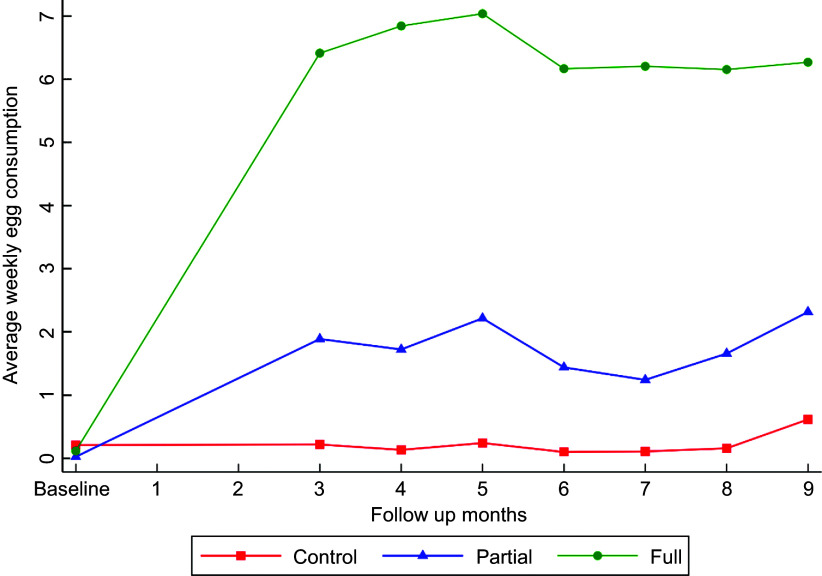
Pattern of egg consumption in follow-up months across research arms.

In egg consumption variables, it was observed that children in the full intervention group consumed eggs more regularly than the partial intervention group and control group. In comparison, very few children consumed eggs in the control group. Average weekly egg consumption was also significantly higher in the full intervention group (6·4) as compared to the partial (1·76) and control group (0·22). Figure 1 shows a pattern of egg consumption across research arms in follow-up months.

Table 2 shows a comparison of average total ASQ score across research arms, and the score was highest in the full intervention group (239·36), as compared with the partial intervention group (223·13) and the control group (223·37). Considering domain-specific outcomes, children in the full intervention group scored higher in almost all developmental domains (except personal social) than the children in the partial or control groups. Independent *t* test showed that children in the full intervention arm had significantly higher mean scores in gross motor, fine motor and problem-solving domains and in overall total scores compared with those in the control. However, in partial intervention arm, no significant difference in the development score was found when compared with the control arm.

**Table 2.** Early child development (ECD) scores by intervention arm

	All (*n* 244)			Full (*n* 78)			Partial (*n* 83)			Control (*n* 83)			Mean difference (*t* test) compared with control arm
Development score	Mean	sd	95 % CI	Mean	sd	95 % CI	Mean	sd	95 % CI	Mean	sd	95 % CI	
Total ASQ scores Range (0–300)	228·4	48·92	222·5, 234·3	239·36	42·6	230, 248·7	223·13	52·3	212·3, 233·9	223·37	49·4	213·1, 233·7	Full: 15·98** Partial: 0·24
Communication total Range (0–60)	48·6	14·2	46·9, 50·4	50·13	12·8	47·2, 53·02	48·25	14·4	45·1, 51·4	47·65	15·1	44·3, 50·9	Full: 2·48 Partial: 0·60
Gross motor Range (0–60)	49·9	9·79	48·6, 51·1	51·73	8·9	49·7, 53·7	49·27	9·0	47·3, 51·2	48·67	11·1	46·3, 51·1	Full: 3·06** Partial: 0·60
Fine motor Range (0–60)	44·5	15·1	42·6, 46·4	47·63	13·5	44·6, 50·7	42·89	16·7	39·2, 46·5	43·1	14·5	39·9, 46·2	Full: 4·56** Partial: 0·18
Problem solving Range (0–60)	40·3	13·69	43·8, 46·5	44·42	12·3	41·7, 47·2	38·61	15·13	35·3, 41·9	38·1	12·7	35·3, 40·8	Full: 6·36*** Partial: 0·54
Person social Range (0–60)	45·1	10·9	43·8, 46·5	45·5	10·8	43, 47·9	44·09	10·9	41·7, 46·5	45·9	10·7	43·5, 48·3	Full: –0·45 Partial: –1·80

ASQ, Ages and Stages Questionnaire.

### Impact of egg consumption on early childhood development

Table 3 reports adjusted odds ratios from logistic regressions (Col. 1–5) and coefficients (*β*) of linear regression models (Col. 6–11). After adjusting for baseline covariates (child’s gender, birth order, child’s anthropometry, mother’s education, mother’s age, household size, wealth index and baseline egg consumption), consistency in egg consumption was found to be statistically significant with lower odds of falling below the cut-off in communication skills ( 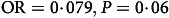 ), gross motor skills ( 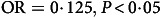 ) and for personal social skills ( 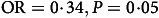 ). Among the covariates, child’s height at baseline is found to be associated with decreasing odds of falling below the cut-off scores in fine motor ( 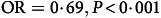 ), problem-solving ( 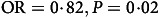 ) and personal social ( 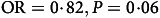 ) domains, and higher weight at baseline was associated with lower odds of falling below the cut-off in communication skills ( 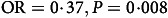 ). The highest wealth index group was associated with a significantly lower odds of falling below the cut-off scores in gross motor ( 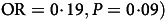 .

**Table 3.** Impact of consistency of egg consumption on ECD

	Logistic regression for falling below the cut-off values						Linear regression for log of total scores				
	Communication	Gross motor	Fine motor	Problem solving	Personal social	Communication	Gross motor	Fine motor	Problem solving	Personal social	Total scores
	(1)	(2)	(3)	(4)	(5)	(6)	(7)	(8)	(9)	(10)	(11)
	OR	OR	OR	OR	OR	*β*	*β*	*β*	*β*	*β*	*β*
Adjusted											
Variables											
Consistency of egg consumption											
1–3 months	0·586	0·335	0·927	0·525	0·857	−0·064	0·015	−0·186	−0·087	−0·000	−0·068
	(0·485)	(0·334)	(0·633)	(0·361)	(0·679)	(0·121)	(0·061)	(0·114)	(0·103)	(0·090)	(0·070)
4–6 months	0·584	0·327	0·864	0·743	0·609	0·020	−0·029	−0·207	−0·071	−0·030	−0·066
	(0·422)	(0·268)	(0·657)	(0·343)	(0·382)	(0·114)	(0·061)	(0·114)	(0·081)	(0·082)	(0·060)
In all months	0·079*	0·125**	1·176	0·608	0·343*	0·050	0·058	−0·044	0·041	0·004	0·007
	(0·106)	(0·112)	(0·795)	(0·334)	(0·190)	(0·087)	(0·052)	(0·088)	(0·069)	(0·078)	(0·040)
Baseline variables											
Birth order	0·766	0·762***	0·882	0·974	0·987	0·014	0·018	0·020	−0·009	−0·007	0·008
	(0·138)	(0·069)	(0·111)	(0·100)	(0·158)	(0·010)	(0·011)	(0·019)	(0·012)	(0·012)	(0·009)
Baseline egg consumption (=1 if yes)	3·134	1·823	1·608	0·838	0·522	−0·011	−0·004	−0·098	−0·031	0·052	−0·013
	(3·794)	(1·949)	(1·609)	(0·698)	(0·543)	(0·069)	(0·052)	(0·110)	(0·080)	(0·055)	(0·045)
Weight (kg)	0·371***	0·777	1·539	1·688*	0·860	−0·014	0·013	−0·035	−0·06**	0·012	−0·017
	(0·138)	(0·365)	(0·428)	(0·473)	(0·310)	(0·039)	(0·026)	(0·037)	(0·025)	(0·026)	(0·017)
Height (cm)	0·849	0·924	0·694***	0·816**	0·821*	0·039***	0·01**	0·010	0·020*	0·015*	0·02***
	(0·117)	(0·077)	(0·047)	(0·073)	(0·085)	(0·011)	(0·004)	(0·010)	(0·010)	(0·007)	(0·005)
Girl	0·775	1·112	1·017	1·063	0·577	0·020	0·030	−0·096	−0·038	0·132***	−0·003
	(0·443)	(0·546)	(0·482)	(0·412)	(0·268)	(0·050)	(0·047)	(0·068)	(0·046)	(0·034)	(0·030)
Mother’s age at first birth	0·802*	1·094	1·115	1·034	0·864	−0·004	−0·009	−0·003	0·001	−0·011	−0·004
	(0·093)	(0·132)	(0·123)	(0·091)	(0·138)	(0·014)	(0·009)	(0·012)	(0·013)	(0·009)	(0·007)
Education of mothers											
Early primary education		2·329	1·062	1·291	1·582	−0·087	0·070	0·151	0·068	−0·059	0·014
		(3·510)	(0·893)	(0·835)	(2·077)	(0·093)	(0·053)	(0·180)	(0·074)	(0·092)	(0·074)
Complimentary primary studies	1·718	1·174			2·608	0·038	−0·162	0·206**	0·104*	−0·169	0·030
	(2·174)	(1·111)			(2·048)	(0·156)	(0·197)	(0·094)	(0·055)	(0·165)	(0·078)
Early secondary education			0·760			0·249***	0·055	0·313**	0·038	0·070	0·143**
			(0·557)			(0·072)	(0·054)	(0·119)	(0·095)	(0·075)	(0·059)
*Kornaic* school					8·724*	−0·121	0·011	0·106	0·079	−0·078	0·004
					(10·13)	(0·128)	(0·059)	(0·100)	(0·068)	(0·082)	(0·025)
HH size											
11–20 persons	0·401	0·555	0·584	1·383	0·944	−0·056	0·019	−0·004	−0·025	0·001	−0·000
	(0·317)	(0·518)	(0·354)	(0·685)	(0·506)	(0·039)	(0·033)	(0·094)	(0·063)	(0·027)	(0·033)
21–30 persons	1·158	2·370	0·941	0·856	1·314	−0·151**	−0·063	−0·038	−0·119	−0·088	−0·067*
	(0·566)	(1·352)	(0·576)	(0·539)	(0·842)	(0·056)	(0·039)	(0·082)	(0·077)	(0·052)	(0·035)
More than 30	0·326	0·461	1·337	0·827		0·105	0·016	0·018	−0·041	0·037	0·033
	(0·360)	(0·451)	(0·885)	(0·481)		(0·081)	(0·034)	(0·111)	(0·077)	(0·037)	(0·049)
Wealth index^*^											−0·000
Middle	0·981	0·949	1·322	1·136	1·466	0·109**	0·021	0·059	−0·051	0·030	0·035
	(0·502)	(0·617)	(0·579)	(0·571)	(0·908)	(0·044)	(0·032)	(0·068)	(0·072)	(0·054)	(0·028)
Highest	0·427	0·193*	0·827	0·973	1·007	0·073	0·046	0·030	−0·023	0·046	0·030
	(0·441)	(0·188)	(0·534)	(0·490)	(0·946)	(0·063)	(0·040)	(0·070)	(0·055)	(0·069)	(0·041)
Constant	3·033e+09**	229·76	1·126e+08***	4144·5*	6 978 048·2**	1·142	3·128***	3·370***	2·840***	2·763***	4·302***
	(2·612e+10)	(1578·018)	(4·573e+08)	(19 736·221)	(47665981·083)	(0·759)	(0·229)	(0·653)	(0·566)	(0·465)	(0·379)
Observations	199	212	214	201	189	234	234	234	230	234	234

ECD, early childhood development.

* Captured only in month 7. *** *P* < 0·01, ** *P* < 0·05, * *P* < 0·1. Clustered se in parentheses.

In multivariate regression models, there was no significant impact of consistency of egg consumption on domain-specific ASQ scores or on total ASQ scores. Among the covariates, as in previous cases, child height at the baseline remained a significant predictor of ASQ scores in children in all domains except problem-solving, with a 1 cm increase in height associated with 1 to 4 % increase in development scores.

In the second stage of the analysis, we tested the quantity of egg consumption per week (Table 4). Weekly egg consumption was not found to be significantly associated with falling below the cut-off scores in any of the domain. Height was a significant predictor of ECD. Height was found to be significantly associated with lower odds of falling below the cut-off in fine motor, problem-solving and personal social skills.

**Table 4.** Impact of quantity of egg consumption on ECD

	Logistic regression for falling below the cut-off values					Linear regression for log of scores					
	Communication	Gross motor	Fine motor	Problem-solving	Personal social	Communication	Gross motor	Fine motor	Problem-solving	Personal social	Total scores
	(1)	(2)	(3)	(4)	(5)	(6)	(7)	(8)	(9)	(10)	(11)
	OR	OR	OR	OR	OR	*β*	*β*	*β*	*β*	*β*	*β*
Adjusted											
Variables											
Average weekly egg consumption	0·798	0·812	1·036	0·928	0·831	0·011	0·005	0·012	0·019**	−0·001	0·008
	(0·121)	(0·107)	(0·08)	(0·074)	(0·094)	(0·008)	(0·006)	(0·01)	(0·008)	(0·01)	(0·005)
Baseline variables											
Birth order	0·780	0·75***	0·885	0·960	0·982	0·014	0·018	0·017	−0·011	−0·007	0·007
	(0·128)	(0·071)	(0·11)	(0·095)	(0·148)	(0·010)	(0·011)	(0·02)	(0·011)	(0·01)	(0·008)
Baseline egg consumption (=1 if yes)	2·358	1·624	1·602	0·764	0·537	−0·022	0·001	−0·104	−0·033	0·054	−0·015
	(2·717)	(1·519)	(1·57)	(0·616)	(0·566)	(0·067)	(0·053)	(0·11)	(0·08)	(0·05)	(0·046)
Weight	0·383**	0·817	1·550	1·710*	0·817	−0·015	0·017	−0·022	−0·056**	0·014	−0·013
	(0·156)	(0·416)	(0·45)	(0·513)	(0·272)	(0·040)	(0·029)	(0·04)	(0·021)	(0·03)	(0·017)
Height	0·853	0·923	0·69***	0·818**	0·826**	0·040***	0·009**	0·008	0·019*	0·015*	0·018***
	(0·109)	(0·082)	(0·05)	(0·080)	(0·077)	(0·010)	(0·004)	(0·01)	(0·010)	(0·01)	(0·005)
Girl	0·651	1·057	1·035	1·125	0·535	0·029	0·030	−0·083	−0·030	0·13***	0·003
	(0·462)	(0·524)	(0·49)	(0·436)	(0·255)	(0·049)	(0·045)	(0·07)	(0·042)	(0·03)	(0·028)
Mother’s age at first birth	0·830	1·064	1·105	1·027	0·864	−0·005	−0·010	−0·008	−0·002	−0·012	−0·006
	(0·126)	(0·137)	(0·11)	(0·084)	(0·142)	(0·014)	(0·009)	(0·01)	(0·013)	(0·01)	(0·007)
Education of mothers											
Early primary education		2·416	1·118	1·376	1·528	−0·077	0·070	0·158	0·064	−0·059	0·017
		(2·974)	(0·92)	(0·809)	(2·028)	(0·090)	(0·051)	(0·18)	(0·074)	(0·09)	(0·073)
Complimentary primary studies	1·415	1·391			2·697	0·054	−0·156	0·23**	0·112*	−0·166	0·039
	(1·787)	(1·375)			(2·233)	(0·153)	(0·199)	(0·09)	(0·061)	(0·17)	(0·084)
Early secondary education			0·760			0·257***	0·056	0·31**	0·035	0·070	0·143**
			(0·55)			(0·069)	(0·049)	(0·11)	(0·089)	(0·07)	(0·055)
* Kornaic* school					8·387**	−0·109	0·022	0·135	0·095	−0·073	0·016
					(8·375)	(0·128)	(0·051)	(0·12)	(0·064)	(0·08)	(0·032)
HH size											
11–20 persons	0·399	0·568	0·584	1·499	0·955	−0·050	0·021	−0·002	−0·026	0·002	0·001
	(0·271)	(0·532)	(0·35)	(0·69)	(0·465)	(0·040)	(0·034)	(0·09)	(0·061)	(0·02)	(0·034)
21–30 persons	1·170	2·720*	0·963	0·939	1·392	−0·148**	−0·062	−0·025	−0·115	−0·087	−0·063*
	(0·527)	(1·550)	(0·53)	(0·540)	(0·854)	(0·057)	(0·039)	(0·08)	(0·073)	(0·05)	(0·033)
>30	0·385	0·557	1·300	0·896		0·106	0·011	0·009	−0·049	0·036	0·029
	(0·471)	(0·626)	(0·87)	(0·542)		(0·074)	(0·035)	(0·11)	(0·076)	(0·04)	(0·048)
Wealth index^*^											
Middle	1·005	0·836	1·330	1·123	1·345	0·112**	0·021	0·050	−0·051	0·029	0·034
	(0·569)	(0·508)	(0·57)	(0·55)	(0·848)	(0·047)	(0·031)	(0·07)	(0·074)	(0·05)	(0·028)
Highest	0·432	0·170*	0·824	0·979	0·956	0·077	0·046	0·019	−0·026	0·045	0·028
	(0·469)	(0·163)	(0·54)	(0·505)	(0·890)	(0·061)	(0·036)	(0·07)	(0·054)	(0·07)	(0·039)
Constant	7·335e+08**	172·273	1·120e+08***	2694·569	6 742 374·560**	1·119	3·17***	3·38***	2·87***	2·8***	4·312***
	(6·182e+09)	(1068·426)	(4·571e+08)	(13 140·27)	(44779377·736)	(0·752)	(0·21)	(0·72)	(0·569)	(0·46)	(0·394)
Observations	199	212	214	201	189	234	234	234	230	234	234

ECD, early childhood development.

* Captured only in month 7.*** *P* < 0·01, ** *P* < 0·05, * *P* < 0·1. Clustered se in parentheses.

For linear regression results (col 6–11), egg consumption was found to be significantly associated with problem-solving skills, where each increase in weekly egg consumption led to 1·9 % increase in scores for problem-solving skills.

**Figure 2.**
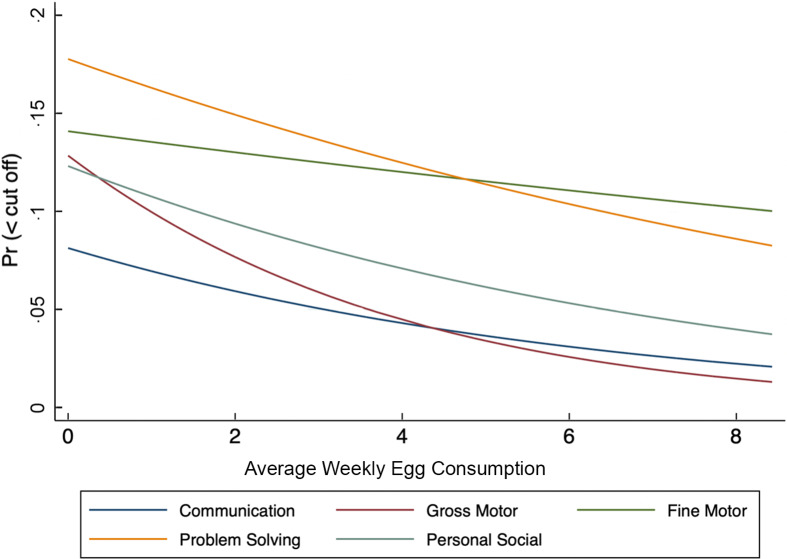
Probability of falling below domain specific ASQ cut-off by weekly egg consumption, ASQ, Ages and Stages Questionnaire.

In Fig. 2, we present the predicted probability of falling below the cut-off scores with respect to average weekly egg consumption based on logistic regressions. Results show that as weekly egg consumption increases, the probability of falling below the cut-off scores for each domain-specific ASQ scores declines. Noticeably, the impact is largest for gross motor, problem-solving and personal social scores, with the steepest decline and with the largest negative change in the probability between 0 and 9 eggs per week corroborating our previous findings with consistency in egg consumption in Table 3.

### Robustness check

#### Propensity score matching

Although we use data from a clustered randomised controlled trial where households in the two treatment arms and the control arm were similar at baseline with no significant differences in egg consumption among children, this study investigates the impact of higher egg consumption resulting from the intervention, not the intervention itself. Thus, there could be a potential of selection bias as the previous model does not account for the determinants of feeding eggs itself and if there are systematic differences between households feeding eggs to the children and those who do not (e.g. more engagement with children and better educated). Therefore, to overcome this potential selection bias, the study uses propensity score matching to estimate the effect of treatment (egg consumption) on ECD. The treatment here is defined as binary and equal to ‘1’ if the respondent reported feeding eggs to the child *in all the 7 months,* and ‘0’ *otherwise*. Propensity scores were generated using a multivariate logistic regression model with egg consumption in all the months as outcome variable and the baseline predictors as follows; produce enough eggs to feed child one egg per day, mother’s age at first birth, mother’s educational status, household size and wealth index. We also checked for some other determinants of feeding eggs related to mother’s knowledge, attitude and practices towards egg consumption. These include knowledge of nutritional importance of eggs, confidence in preparing eggs, difficulty in getting eggs, whether received nutritional knowledge about eggs, etc., but these factors did not appear to be statistically significant of feeding eggs, therefore were dropped from the model. Propensity score matching one to one matching was performed using nearest neighbour matching. The matching ensures that groups of households who fed their children and those who did not were systematically identical. For brevity (matching results are available on request)), here we only report the results for average treatment effect on treated in Table 5. The coefficients in Table 5 indicate the difference in the scores among the treatment group (children who ate eggs in all the months) due to the treatment (egg consumption in all months). The significant increase in gross motor skills and fine motor skills aligns with our previously reported results in Table 3. However, we do not find significant increase in communication and personal social skills. Hence, the findings confirm the significant contribution of egg consumption on ECD outcomes after ensuring selection issues were controlled for.

**Table 5.** Effect of egg consumption on child development outcomes – average treatment effect on the treated (ATET)

	Coefficient	Clustered se	95 % CI
Total scores	0·093**	0·037	0·0198, 0·1670
Communication	0·094	0·069	−0·0412, 0·2300
Gross motor	0·106*	0·056	−0·0031, 0·214
Fine motor	0·134**	0·065	0·006, 0·262
Problem-solving	0·059	0·057	−0·054, 0·171
Personal social	0·057	0·057	−0·056, 0·169

** *P* < 0·05, * *P* < 0·1.

## Discussion

The empirical analysis in this article provides supporting evidence for the promotion and inclusion of eggs in child diet to improve ECD. The challenges of food insecurity and malnutrition have been directly linked to the nutrient deficiencies and their contribution to learning and development deficits^(32)^. Delays in ECD contribute to cognitive and motor development deficits, low educational attainment and intergenerational transmission of poverty^(33)^. Addressing malnutrition, especially in early childhood, is critical to the prevention of poor cognitive and health outcomes. Findings highlighted the impact of full intervention and partial intervention as part of *Un Oeuf* trial on ECD scores. Children in the full intervention group who received chickens and BCC package reported highest ASQ scores in almost all domains (except personal social) than the children in partial intervention group (who only receive the BCC package) and the control group. Though the difference across partial and control group was not statistically significant indicating the importance of accessibility and availability of eggs over behaviour change communication. Furthermore, the impact of eggs consumption in improving ECD is highlighted with this study with implications in LMIC.

The key findings of the paper hold statistical significance after baseline adjustment for three domains (communication, gross motor and personal social) for consistency of egg consumption and for one domain (problem-solving) for quantity of egg consumption. Result showed that each additional egg per week led to a 1·9 % increase in problem-solving scores in children establishing a dose–response relationship between egg consumption and problem-solving skills scores.

Another important result was surrounding consistency of egg consumption. We find that children who consumed eggs consistently in all months (7) of intervention period were significantly less likely to fall below the cut-off score for communication, gross motor and personal social skills, all of which are crucial elements of overall child development.

Our findings are consistent with Miller *et al.* (2020), who found egg consumption to be associated with lower odds of having ASQ score in the bottom quartile in Nepal. A study in Ethiopia also reported that children who had increased egg intake attained gross motor skills at a significantly earlier age compared with controls^(34)^. Similar to ours, the study by Omer *et al.* (2022) focused on child owned poultry as compared to some other trials that distributed eggs directly to the children^(22,24)^. Similarly, a meta-analysis also found that micronutrients played a crucial role in cognitive performance among children aged 6–11 years^(35)^, which suggests a possible mechanism underlying the findings reported here.

Our findings show that consistent consumption of eggs led to improved ECD in the domains of communication, gross motor and personal social. Eggs contain numerous nutrients that play a vital role in neurocognitive development including Fe, Zn, choline, folate, iodine and long-chain PUFA such as docosahexanoic acid^(36)^. The improved nutritional status as manifested by decreased wasting and underweight^(19)^ among the full intervention group might have also contributed to better ECD. Nutritional status is found to have significant contribution on gross motor and fine motor skills though the findings have been mixed and not strong and consistent^(37,38)^. Improved anthropometry is significantly associated with motor development and language skills^(39)^. Additionally previous studies^(7)^ have noted significant association of motor development with executive functions, memory and later cognitive development in children. Therefore, the results of the study indicate the importance of better childhood nutrition and underscoring eggs as a complementary food option.

In Burkina Faso, undernutrition is one of the leading causes of morbidity in children-under-five ^(40)^. Behavioural and nutrition interventions, such as the *Un Oeuf* project, can be effective among vulnerable population for improved decision-making, enhancing knowledge of nutrition, and improving access to livestock production resources. However, there are prevalent gaps in research as more information is needed to find appropriate and effective approaches to improve children’s developmental and nutritional status at specific intervals of childhood development. Research conducted by Miller *et al.* (2020) states nutritional intervention not only affect the development outcomes immediately but also may be evident and permanent later in life. Thus, the extension of the study can be done by following the children in the later stages of childhood and adolescence and check how and if the effect of intervention continued over time.

This study also has a few limitations. First, ASQ-3 has its own reported rules^(41)^ in capturing information and indicators of child development, including a stated purpose as a screening tool designed to recognise developmental delays but not affirm child development status. Like others who have used or modified the ASQ for research in LMIC, the research team believes that the ASQ provides meaningful information about different domains of development in children^(28,30,42)^. Second, there could have been unique distractions or curiosities for children completing the evaluation that led to differences or biases in their recorded responses. For example, some children in these villages never had access to a mirror. Thus, for many, it was their first time seeing their reflection and could impact the ability to accurately assess childhood development. Third, as this study was a behaviour change intervention, there is possibility of social desirability bias in reporting egg consumption especially in the full intervention group, although the questionnaire was administered to control for the same. Additionally, the weekly egg consumption measure is based on a 7-day dietary recall with a potential to recall bias in the dataset. Finally, other variables, such as environmental exposure, illness, other diet, maternal factors and water and sanitation, could have influenced child development during the study period and were not directly measured in our study.

### Conclusion

ECD is closely related to the diets and nutritional status of children. Leveraging data collected during and just after the *Un Oeuf* intervention in Burkina Faso, this study found that both consistency and quantity of egg consumption led to observed improvements: greater consistency of egg consumption led to lower odds of falling below the ASQ-3 cut-off score for gross motor, personal social skills and the total development score. The study also found that increase in the quantity of egg consumption led to an increase in scores in problem-solving domain. These results underscore the importance of further research on dietary interventions in LMIC, with particular focus on complexity of nutrition as well as culturally appropriate tools that can measure child development in low-resource settings in LMIC. Given the relative affordability and accessibility of eggs, integrating them into nutrition-focused public health programmes could serve as a feasible and effective strategy to improve ECD outcomes in these countries.

## Supporting information

Ernyey et al. supplementary material Ernyey et al. supplementary material

## Supplementary material

For supplementary material accompanying this paper visit https://doi.org/10.1017/S1368980024002490
