# Supplementary material for: Effect of egg consumption on early childhood development: evidence from Un Oeuf study
Source: Public Health Nutr. 2024 Dec 12;28(1):e10. doi: 10.1017/S1368980024002490 (PMC11736653; doi:10.1017/S1368980024002490)
Supplement: Ernyey et al. supplementary material [file S1368980024002490sup001.docx]

Appendix 1: Impact of consistency of egg consumption on ECD

|  | Logistic regression for falling below the cut-off values | | | | | Linear regression for log of total scores | | | | | |
| --- | --- | --- | --- | --- | --- | --- | --- | --- | --- | --- | --- |
|  | Communication | Gross Motor | Fine Motor | Problem Solving | Personal Social | Communication | Gross Motor | Fine Motor | Problem Solving | Personal Social | Total Scores |
|  | (1) | (2) | (3) | (4) | (5) | (6) | (7) | (8) | (9) | (10) | (11) |
|  | OR | OR | OR | OR | OR | β | β | β | β | β | β |
| Unadjusted | | | | | | | | | | |  |
| **Variables** |  |  |  |  |  |  |  |  |  |  |  |
| *Consistency of Egg Consumption* | | | | | | | | | | | |
| 1-3 months | 0.805 | 0.361 | 0.756 | 0.694 | 0.844 | -0.049 | -0.028 | 0.024 | -0.128 | -0.083 | -0.049 |
|  | (0.578) | (0.277) | (0.559) | (0.357) | (0.514) | (0.069) | (0.116) | (0.057) | (0.134) | (0.093) | (0.069) |
| 4-6 months | 0.778 | 0.225** | 0.836 | 0.758 | 0.686 | -0.035 | 0.043 | -0.007 | -0.126 | -0.040 | -0.035 |
|  | (0.607) | (0.167) | (0.496) | (0.290) | (0.377) | (0.054) | (0.109) | (0.064) | (0.102) | (0.061) | (0.054) |
| In all months | 0.111** | 0.083*** | 0.616 | 0.556 | 0.422 | 0.049 | 0.101 | 0.086* | 0.045 | 0.072 | 0.049 |
|  | (0.115) | (0.063) | (0.372) | (0.276) | (0.232) | (0.042) | (0.082) | (0.044) | (0.099) | (0.051) | (0.042) |
| Constant | 0.107*** | 0.292** | 0.192*** | 0.240*** | 0.148*** | 5.417*** | 3.788*** | 3.854*** | 3.773*** | 3.666*** | 5.417*** |
|  | (0.041) | (0.149) | (0.101) | (0.071) | (0.062) | (0.035) | (0.073) | (0.041) | (0.092) | (0.042) | (0.035) |
| Observations | 244 | | | | | 243 | | | | | |

Appendix 2: Impact of quantity of egg consumption on ECD

|  | Logistic regression for falling below the cut-off values | | | | | Linear regression for log of scores | | | | | |
| --- | --- | --- | --- | --- | --- | --- | --- | --- | --- | --- | --- |
|  | Communication | Gross Motor | Fine Motor | Problem Solving | Personal Social | Communication | Gross Motor | Fine Motor | Problem Solving | Personal Social | Total Scores |
|  | (1) | (2) | (3) | (4) | (5) | (6) | (7) | (8) | (9) | (10) | (11) |
|  | OR | OR | OR | OR | OR | β | β | β | β | β | β |
| Unadjusted | | | | | | | | | | |  |
| **Variables** |  |  |  |  |  |  |  |  |  |  |  |
| *Average weekly egg consumption* | 0.844 | 0.751* | 0.955 | 0.901 | 0.858* | 0.015 | 0.008 | 0.018* | 0.022*** | 0.001 | 0.012* |
|  | (0.098) | (0.119) | (0.06) | (0.069) | (0.070) | (0.010) | (0.005) | (0.01) | (0.006) | (0.01) | (0.006) |
| Constant | 0.088*** | 0.147*** | 0.16*** | 0.216*** | 0.140*** | 3.79*** | 3.867*** | 3.67*** | 3.598*** | 3.78*** | 5.380*** |
|  | (0.050) | (0.045) | (0.04) | (0.050) | (0.044) | (0.055) | (0.020) | (0.04) | (0.025) | (0.03) | (0.03) |
| Observations | 244 | | | | |  | | | | | 243 |
